# The Characterization of Lactic Acid Bacteria Strains as Components of a Biopreparation for Chickens for Slaughter

**DOI:** 10.3390/microorganisms13020317

**Published:** 2025-02-01

**Authors:** Daria Zamojska, Justyna Rosicka-Kaczmarek, Ewa Macierzyńska-Piotrowska, Adriana Nowak

**Affiliations:** 1 Polwet Sp. z o.o., M. Konopnickiej 21, 98-100 Lask, Poland; dariazie75@gmail.com (D.Z.);; 2 Institute of Food Technology and Analysis, Faculty of Biotechnology and Food Science, Lodz University of Technology, Stefanowskiego 2/22, 90-530 Lodz, Poland; 3 Department of Environmental Biotechnology, Faculty of Biotechnology and Food Sciences, Lodz University of Technology, Wolczanska 171/173, 90-530 Lodz, Poland

**Keywords:** probiotic, probiotic properties, antagonistic activity, broiler chicken, organic acids, whey medium

## Abstract

Since 2022, the European Union has banned the use of antibiotics in animal production. We conducted studies to characterize *Lactiplantibacillus plantarum* (47, AN8, and OK-B) and *Ligilactobacillus salivarius* (AN9) and evaluate their potential to create a biopreparation based on fermented whey for chickens. The following methods were used: lactic acid bacteria (LAB) culture and storage, crystal violet staining, Koch’s plate method, Caco-2 cell culture, hydrophobicity test, and spectrophotometric measurements. All bacteria showed weak adhesion to polystyrene and collagen, and the *L. plantarum* species demonstrated weak adhesion to mucus. All bacteria showed strong adhesion to the intestinal epithelial cell line Caco-2. LAB showed strong autoaggregation and coaggregation with *E. coli* ATCC10536. The highest affinity for xylene was exhibited by *L. salivarius* AN9 (above 30%) while, for chloroform, the highest affinity was exhibited by *L. plantarum* OK-B (approx. 95%); the affinity for n-hexadecane for all strains was below 20%. The highest survival in the presence of bile salts (0.3%) was demonstrated by *L. plantarum* 47 (above 54%). The effect of low pH resulted in decreased viability for all strains. Significant differences were demonstrated in the concentration of lactic acid between MRS and whey medium after culturing LAB. These results will aid in qualifying these strains for further research to create a functional feed for chickens.

## 1. Introduction

Using sub-therapeutic amounts of antibiotic growth promoters (AGPs) in feed enhances its digestibility and increases body weight in animal farms [1,2]. The addition of AGPs reduces the occurrence of pathogenic bacteria that can cause individual losses during breeding while also eliminating beneficial lactic acid bacteria (LAB) that are essential to the intestinal microbiota of chickens [3,4]. In animals treated with antibiotics, a positive effect on the feed conversion ratio (FCR) has been observed; unfortunately, this also resulted in the simultaneous appearance of heterotrophic bacteria resistant to antibiotics in fecal samples [5]. In research on new natural substitutes for antibiotics, scientists strive to obtain a low FCR, which will have a positive economic impact [6]. The increasing incidence of negative effects from antibiotic use on animal farms and the European Union’s ban on the prophylactic use of antibiotics has prompted the search for alternative natural substances to use as a functional food with antimicrobial properties that can hopefully help to stabilize the intestinal microbiome of chickens and stimulate their immune system [7,8,9]. In 2023, poultry production was the second largest meat production sector (13.3 mln ton) after pork production. Poland is the main country responsible for poultry farming [10]. Because of this, the search for natural substitutes for antibiotics is becoming increasingly important. One such substitution may be probiotic preparations, which contribute to maintaining the balance of intestinal microorganisms and, similarly to antibiotics, inhibit the growth of pathogenic microorganisms in farm animals. According to the definition used by the International Scientific Association for Probiotics and Prebiotics (ISAPP), probiotics are “live microorganisms that, when administered in adequate amounts, confer a health benefit on the host” [11,12].

According to the FAO [13], for a given bacterial strain to be classified as probiotic and used in animal breeding, it must meet several criteria, including the following [13,14,15,16,17]: not contain drug resistance genes; be nonpathogenic to the host; demonstrate high survival in the gastrointestinal environment (low pH and the presence of bile acids); demonstrate the ability to adhere to intestinal epithelial cells; maintain viability from transport to storage to application; positively modulate the gut microbiome; improve digestion and absorption of nutrients; produce substances with antimicrobial activity (which must be proven); and, finally, the type, species, and strain should be precisely defined and its name should be indicated on the given probiotic product.

The type of substances used to produce biopreparations may be prebiotics. According to the ISAPP, a prebiotic is a “substrate selectively used by microorganisms, bringing a beneficial effect on the health of the host” [18,19]. Prebiotics should primarily have a positive effect on the gastrointestinal tract and, additionally, lower the level of lipids in the blood or strengthen osteocytes by increasing the bioavailability of minerals [20,21,22]. In poultry farming, the most commonly used feed additives include inulin, yeast extract, lactulose, and galactooligosaccharides (GOS), which are prebiotics [23].

A product containing a combination of probiotics and prebiotics is called a synbiotic [24]. The use of synbiotics as a feed additive and functional food product can reduce the occurrence of campylobacteriosis in livestock farming, which can combat this disease among humans [25]. Postbiotics can also be used as a substitute for ASW in chicken farming. According to the latest ISAPP definition, a postbiotic is a “preparation of non-living microorganisms and/or their components that confers a health benefit to the host” [12]. Postbiotics can be an alternative approach to reducing the number of foodborne pathogens and extending the shelf life of poultry meat [26,27]. LAB, during growth, produce or release soluble substances with diverse activity after the lysis of bacterial cells. Some of these substances, including organic acids, bacteriocins, carbon dioxide, diacetyl, fatty acids, and hydrogen peroxide, are characterized by antibacterial activity [28]. Organic acids such as lactic acid and short-chain fatty acids (SCFAs, e.g., acetic acid) inhibit the growth of pathogenic bacteria found in animal husbandry [29,30,31]. The supplementation of feed or water with organic acids improves growth, FCR, meat quality, and intestinal morphology in chickens for fattening [32,33].

Pathogens of chickens for slaughter are most commonly found in large-scale farms and are a significant problem, which is why it is important to develop innovative methods for their elimination; for example, by using probiotic biopreparations with enhanced antibacterial activity against pathogens. In light of the above, the main aim of this study was to characterize LAB strains in terms of their probiotic potential, providing information that can serve as the basis for developing an innovative biopreparation intended for chickens for slaughter and so reduce the use of antibiotics. In this work, some probiotic features of LAB strains, such as cell wall characteristics, were assessed by testing adhesion to biotic (mucus, collagen, and Caco-2) and abiotic (polystyrene) surfaces, hydrophobicity, autoaggregation, and coaggregation with *E. coli* ATCC 10536. Additionally, a test was conducted to check the viability of LAB strains in a simulated gastrointestinal tract (in vitro). The concentration of lactic acid was determined after culturing the bacteria on a whey medium in comparison to the standard medium.

## 2. Materials and Methods

### 2.1. Bacterial Strains and Growth Conditions

We used lactic acid bacteria (LAB) derived from the Pure Culture Collection of the Institute of Fermentation Technology and Microbiology (ŁOCK 105), Lodz University of Technology, and from our collection at the Department of Environmental Biotechnology, Lodz University of Technology. These included *Lactiplantibacillus plantarum* OK-B, 47 (from plant silage), and AN8, and *Ligilactobacillus salivarius* AN9 (from chicken feces). The pathogenic strain *Escherichia coli* ATCC 10536 was purchased at Argenta Sp. z o.o., Poznań, Poland, September 2020, KWIK-STIK™. The above strains were stored at −80 °C on a ceramic bead before use. The LAB strains were activated by transferring the frozen bacteria on the ceramic bead to an MRS medium, where they were incubated for 24 h at 30 °C. The pathogenic strains were activated by transferring the frozen bacteria on the ceramic bead to a broth enriched with 2% glucose, followed by incubation for 24 h at 37 °C. In addition, the LAB strains, after activation in the MRS medium, were cultured in a whey medium (1% whey, 0.5% skimmed milk, and 1% yeast extract). The inoculum in a volume of 5% (*v*/*v*) was added to the MRS liquid medium and incubated for 24 h at 30 °C. The cells were subjected to two passages on the medium based on whey powder under the following conditions: 2% inoculum, incubated for 24 h at 30 °C.

### 2.2. Preparation of Cell-Free Supernatants (CFSs) After LAB Culture in MRS or Whey Medium

The LAB strains were activated according to the procedure described in Section 2.1, and 3 passages were carried out. Then, the 16 h cultures were centrifuged (13,585× *g*/min, 22 °C, 10 min). The supernatant was collected and filtered twice sequentially through filters (Sterile Syringe Filter PVDF/L) with a pore diameter of 0.45 µm and 0.22 µm. CFSs prepared this way were frozen and stored at −80 °C until analysis. CFSs were treated in the same way after culturing LAB on whey medium.

### 2.3. Characterization of the Cell Wall Surface

#### 2.3.1. Adhesion of LAB Strains to Biotic and Abiotic Surfaces

LAB strains were activated using the procedure described in Section 2.1. One passage was performed by adding the inoculum in a volume of 5% (*v*/*v*) to the MRS liquid medium. After 24 h, the liquid cultures were subjected to two centrifugations (3864× *g*/min, 10 min). Then, the supernatant was removed, and the cell pellet was suspended in a PBS solution so that the absorbance value for this solution was equal to 1.0 ± 0.1 for the wavelength λ = 600 nm. Cell suspensions prepared this way were used to determine the adhesion properties to selected abiotic (polystyrene) and biotic (collagen, mucus, and Caco-2 cell line) surfaces.

##### To Polystyrene

Adhesion to polystyrene was performed according to the modified methods described by Tsai et al. (2021), Leccese et al. (2020), and Wang, Da et al. (2020) [34,35,36]. Bacterial cell suspensions in PBS (A_600_ = 1.0 ± 0.1) were applied to a 96-well polystyrene plate (Greiner Bio-One, Kremsmünster, Austria, LOT: E2000838V) with a flat bottom. PBS was used as the negative control. The plate was incubated for 2 h at 37 °C. After this, non-adhered bacterial cells were aspirated. To fix the adhered cells, 80% methanol was added to cover the bottom of each well. The methanol was aspirated, and the cells were stained with 0.1% crystal violet. Incubation was carried out for 15 min at room temperature. After washing twice with PBS, 30% acetic acid was added to the wells and incubated for 15 min on an orbital shaker (100–120 rpm/min) to extract violet from the adhered bacteria. Absorbance was measured using a microplate reader (Berthold Technologies, Bad Wildbad, Germany) at a wavelength of λ = 630 nm. The following formula was used for calculations:

A = A_sample_/A_control_.

Adhesion was classified as follows [37]: A ≤ 1—no adhesion; 2 ≥ A > 1—weak adhesion; 3 ≥ A > 2—medium adhesion; and A > 3—strong adhesion.

##### To Mucus

The adhesion of LAB to mucus was assessed according to a modified method described by Styková et al. (2013) [38]. Pig stomach mucus (150 mg/mL) in PBS at pH 7.2 was sterilely applied to a 96-well flat-bottom plate and incubated for 72 h at 4 °C to bind to the polystyrene surface. After this, the unbound mucus was carefully aspirated and fixed for 20 min at 60 °C. The prepared bacterial cell suspensions in PBS (A_600_ = 1.0 ± 0.1) were applied to the wells of the plate coated with mucus. PBS was used as the negative control. The plate was incubated for 2 h at 37 °C. After incubation, non-adhered bacterial cells were aspirated, and the wells were washed with PBS and aspirated. In order to fix the adhered bacterial cells, the plates were incubated for 20 min at 60 °C. Then, 0.1% crystal violet was added to each well and incubated for 15 min at room temperature. The violet was aspirated, and the wells were washed with PBS. To extract crystal violet from the adhered cells, 20 mM citrate buffer at pH = 4.3 was added and incubated for 45 min at room temperature on an orbital shaker (150 rpm/min). The samples were transferred to a new, clean plate, and the absorbance was measured at a wavelength of λ = 570 nm. Calculations were performed according to the formula in “To polystyrene”. 

##### To Collagen

LAB adhesion to collagen was assessed using the modified methods described by Enriquez-Verdugo et al. (2004) and Maddocks et al. (2013) [39,40]. Bacterial cell suspensions in a PBS solution (A_600_ = 1.0 ± 0.1) were applied to a 96-well, ready-made Type I collagen-coated plate (BioCoat ^®,^ Horsham, PA, USA) via a Corning producer with a flat bottom. The PBS was used as a control. The procedure was then carried out as described in “To polystyrene”.

##### To Intestinal Epithelial Cell Line Caco-2

The intestinal epithelial cell line Caco-2 (purchased at Cell Line Service GmbH, Eppelheim, Germany, November 2014, passage 41) was cultured according to the methodology described by Nowak et al. (2022) [41]. The culture medium was high-glucose DMEM with 10% FBS, 4 mM GlutaMAX^TM^, 25 mM HEPES, mixtures of 100 µg/mL streptomycin, and 100 IU/mL penicillin. Cells were incubated for 7 days at 37 °C in the presence of 5% carbon dioxide and 95% humidity. Every 3 days, the cells were washed with PBS, and the medium was changed. LAB adhesion to the intestinal epithelial cell line Caco-2 was performed according to the modified methods described by Rocha-Mendoza et al. (2020) and Wang, Da et al. (2020) [36,42]. LAB cells were prepared according to the procedure described in Section 2.1 and suspended in a DMEM culture medium without antibiotics and FBS. The bacterial suspension was applied to a 24-well plate with a previously cultured monolayer of Caco-2 cells. The plate was incubated for 2 h at 37 °C in the presence of 5% carbon dioxide. After incubation, DMEM was removed from the cells together with non-adhered bacteria and washed twice with PBS. To detach Caco-2 cells from the surface, trypsin was applied and incubated for 10–15 min at 37 °C. PBS was added, and samples were transferred to Eppendorf tubes and centrifuged (3864× *g*/min, 10 min, 22 °C). Then, the supernatant was removed, and the pellet was suspended in 0.1% Triton X-100 and incubated for 5 min at room temperature to lyse Caco-2 cells. Finally, plating was performed on Petri dishes using Koch’s plate method. Adhesion to Caco-2 cells was not directly tested due to cell lysis. The study measured the sum of bacteria adhering to and penetrating the cells.

#### 2.3.2. Hydrophobicity Testing

Hydrophobicity determination was performed based on the modified methods described by Jena et al. (2013) and Barzegar et al. (2021) [43,44]. LAB strains were prepared for testing in the same way as in Section 2.3.1. An amount of 1 mL of one of three organic solvents was added to the bacterial cell suspension—n-hexadecane, xylene, or chloroform—and vortexed for 30 s to form an emulsion. Then, a 60 min incubation at room temperature was carried out, after which 2 mL of cell suspension was taken in PBS. The n-hexadecane and xylene were placed under the solvent, while chloroform was placed above it; then, the absorbance of the sample was measured at a wavelength of λ = 600 nm. The experiment was performed in 3 replicates from 3 parallel cultures. Hydrophobicity was calculated according to the following formula:

$$Hydrophobicity\left[\%\right]=\left[\frac{A_{0}-A_{t}}{A_{0}}\right]\times 100\%,$$

where

A_0_—initial absorbance;
A_t_—absorbance after 60 min.

#### 2.3.3. Autoaggregation

The autoaggregation of LAB was performed based on the modified method described by Barzegar et al. (2021) [44]. LAB strains were prepared for the study in the same way as given in Section 2.3.1. Bacterial suspensions were left for 24 h at room temperature. After this time, samples were taken, and absorbance was measured at a wavelength of λ = 600 nm. The experiment was performed in 3 replicates from 3 parallel cultures. The following formula was used for calculations:

$$Autoaggregation\left[\%\right]=\left[\frac{A_{0}-A_{t}}{A_{0}}\right]\times 100\%,$$

where

A_0_—initial absorbance;
A_t_—absorbance after 24 h.

#### 2.3.4. Coaggregation of Selected LAB with *E. coli* ATCC 10536

Coaggregation was performed based on the modified method described by Chlebicz-Wójcik et al. (2020) [45]. Then, 24 h cultures of LAB and *E. coli* ATCC 10536 were centrifuged (3864 × *g*/min, 10 min), and the supernatant was removed. The pellet was then washed and suspended in PBS. At a wavelength of λ = 600 nm, the absorbance value was determined to be 0.8 ± 0.1. The suspensions prepared this way were mixed in a 1:1 ratio (pathogen/LAB). The suspensions were left for 24 h at room temperature. After this, 2 mL of the sample was collected, and the absorbance was measured at a wavelength of λ = 600 nm. The experiment was performed in 3 replicates from 3 parallel cultures. The following formula was used for calculations:

$$Coaggregation\left[\%\right]=\frac{\left(\frac{Ax+Ay}{2}\right)-A(x+y)}{\frac{Ax+Ay}{2}}\times 100,$$

where

A_x_—initial absorbance of LAB;
A_y_—initial absorbance of the pathogen;
A_(x+y)_—absorbance after 24 h of the pathogen/LAB suspension.

### 2.4. Survival of LAB in the Presence of Bile Salts and Low pH

The survival of LAB in the presence of bile salts was performed according to the modified method described by Cizeikiene et al. (2021) [46]. LAB were activated according to Section 2.1, and 2 passages were performed. After 24 h, the cultures were centrifuged (3864× *g*/min, 10 min) and then suspended in physiological saline (0.85%). The control was physiological saline without bile salts. The sample contained 0.3% (*w*/*v*) bile salts. The suspensions prepared in this way were incubated at 40 °C. An appropriate dilution (using the Koch’s plate method) was plated with 0, 1, 2, and 4 h of incubation. The plates were incubated for 48 h at 30 °C. The effect of bile salts on LAB was determined based on the colonies grown on MRS agar plates at different incubation times at 40 °C [47] and expressed as percentage survival. The tests were performed in duplicate from 3 parallel cultures.

LAB survival at low pH was performed according to the modified method described by Nemska et al. (2019) and Benbara et al. (2020) [14,48]. LAB strains (3% *v*/*v*) were cultured for 24 h at 30 °C, then centrifuged (3864× *g*/min, 10 min) and washed with PBS buffer. The bacterial pellet was suspended in PBS, divided into two samples, and centrifuged again (3864× *g*/min, 10 min). One part of the pellet was suspended in PBS buffer (control), and the other in PBS buffer at pH = 2 (determined using HCl) at a final cell number of 10^8^–10^9^ cfu/mL and incubated for 4 h at 40 °C. Appropriate dilutions (using Koch’s plate method) were plated using the plate method from 0, 1, 2, and 4 h of incubation. The plates were incubated for 48 h at 30 °C. The effect of low pH on LAB was determined based on the colonies grown on MRS agar plates at different incubation times at 40 °C and expressed as a survival percentage. The tests were performed in duplicate from 3 parallel cultures.

### 2.5. Quantification of the Profile of Lactic Acid in CFSs

CFSs after LAB culture in MRS and whey medium were prepared according to Section 2.2. The control consisted of pure MRS medium and pasteurized whey medium. Quantification of the profile of lactic acid in CFSs was performed according to a modified method presented by Chen et al. (2019) [49]. The chromatographic separation was determined according to the studies conducted by Nowak et al. (2022) [41]. All measurements were performed in 3 repetitions from 3 parallel cultures. Direct identification was made by analyzing the characteristic retention time for lactic acid. The concentration was determined by calculating the area under each peak and the acid calibration curve.

### 2.6. Statistical Methods

The following statistical tests were used to analyze the data:

- Simple classification, Tukey’s test: autoaggregation of LAB strains; differences between pairs of “LAB strain and *E. coli* ATCC 10536”; and LAB hydrophobicity (xylene);
- Dunn’s test: LAB hydrophobicity (chloroform and n-hexadecane); and the differences between the concentrations of lactic acid;
- Kruskal–Wallis test: adhesive properties of LAB to biotic and abiotic surfaces;
- Two sample *t*-tests: adhesion to Caco-2 cells; coaggregation of LAB strains and *E. coli* ATCC 10536; differences between the concentration of lactic acid obtained in post-fermentations after LAB cultivation on the MRS medium and whey medium; and the effect of bile salts and low pH on LAB viability.

Calculations were made using an online calculator [50].

## 3. Results

### 3.1. Adhesive Properties of LAB Strains to Biotic and Abiotic Surfaces

**Figure 1:**
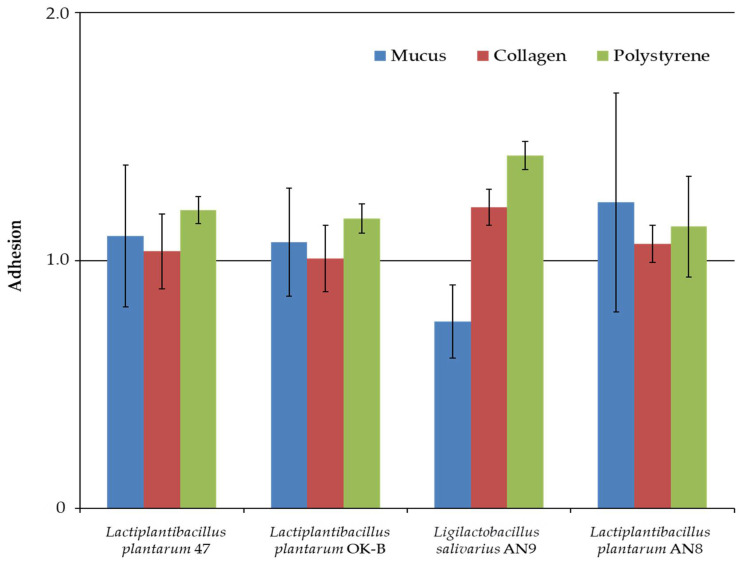
Adhesion of LAB strains to abiotic (polystyrene) and biotic (collagen and mucus) surfaces after 2 h of incubation. Experiments were performed in three independent experiments and presented as mean ± standard deviation.

According to the classification adopted in Section 2.3.1, results above 1 indicate weak adhesion, and anything below this value indicates no adhesion to the selected surface. Weak adhesion to mucus was demonstrated by *L. plantarum* 47, OK-B, and AN8; in the remaining strains, this ability was not observed (Figure 1). Weak adhesion to collagen was only observed for *L. salivarius* AN9. All of the strains also showed weak adhesion to polystyrene.

Measurements of adhesion to collagen showed differences between *L. plantarum* 47 and *L. salivarius* AN9. In the case of adhesion to polystyrene, significant differences were shown between *L. salivarius* AN9 and *L. plantarum* AN8 (Kruskal–Wallis test, for *p* < 0.01).

**Figure 2:**
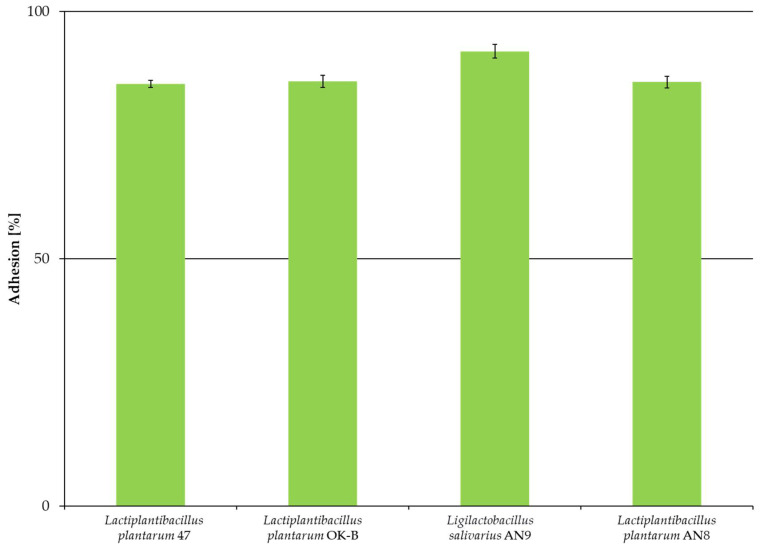
Adhesion of LAB strains to the Caco-2 cell line after 2 h of incubation. The experiment was performed in three independent replicates and results are presented as mean ± standard deviation.

The graph below (Figure 2) presents the adhesion (in %) of the four tested LAB strains to the model intestinal epithelial cell line Caco-2.

Based on the obtained results (Figure 2), it can be stated that all strains showed strong adhesion to Caco-2 cells (above 85%), but no significant statistical differences were found between strains based on simple classification (*p* < 0.05). The strongest adhesion ability was shown by *L. salivarius* AN9 (91.95% ± 1.34%) and the weakest by *L. plantarum* 47 (85.34% ± 1.38%). Based on Student’s *t*-test for paired samples (Table 1), differences were found between the values of LAB adhesion to the Caco-2 cell line compared to the control, which was the density of bacteria before adhesion to the Caco-2 cell line.

### 3.2. LAB Cell Wall Properties: Autoaggregation, Coaggregation, and Hydrophobicity

**Figure 3:**
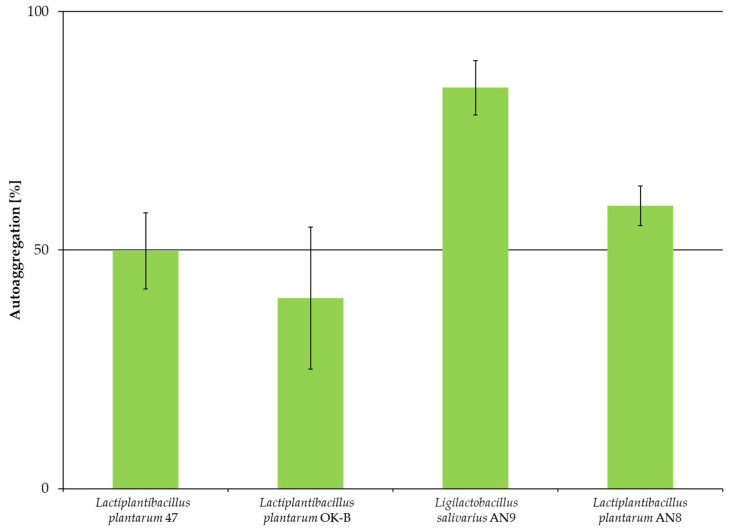
Autoaggregation ability of LAB after 24 h of incubation. Experiments were performed in three independent replicates and presented as mean ± standard deviation.

In this study, the autoaggregation capacity of four LAB strains was assessed: *L. plantarum* 47, *L. plantarum* OK-B, *L. salivarius* AN9, and *L. plantarum* AN8. Figure 3 shows the degree of autoaggregation (in %) of individual strains after 24 h of incubation.

The obtained results (Figure 3) allowed us to determine that the highest degree of autoaggregation was obtained for *L. salivarius* AN9 (approximately 84%) and the lowest for *L. plantarum* OK-B (approximately 40%). Two strains (*L. salivarius* AN9 and *L. plantarum* AN8) showed autoaggregation above 50%. Simple classification and Tukey’s test (*p* < 0.01) were used to test for the statistical significance between autoaggregation values for individual LAB strains (Table 2).

**Figure 4:**
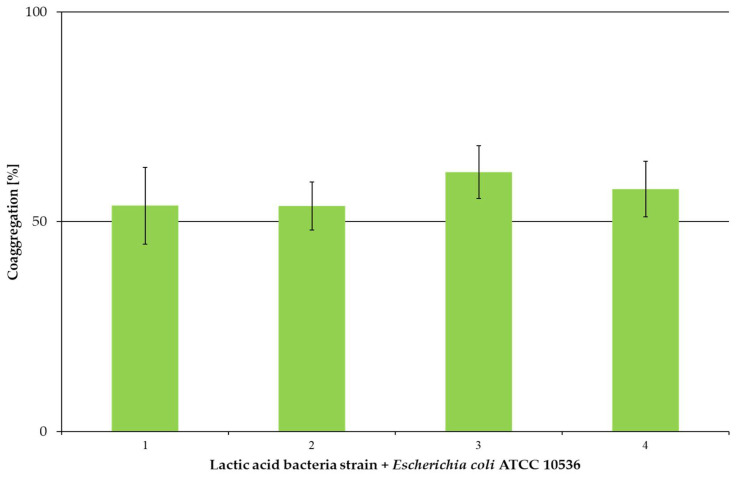
Coaggregation of LAB with *Escherichia coli* ATCC 10536 after 24 h of incubation. Experiments were performed in three independent replicatesand result are presented as mean ± standard deviation. 1: *Lactiplantibacillus plantarum* 47 + *Escherichia coli* ATCC 10536; 2: *Lactiplantibacillus plantarum* OK-B + *Escherichia coli* ATCC 10536; 3: *Ligilactobacillus salivarius* AN9 + *Escherichia coli* ATCC 10536; 4: *Lactiplantibacillus plantarum* AN8 + *Escherichia coli* ATCC 10536.

Statistical analysis showed differences between strains at *p* < 0.01. *L. plantarum* AN8 did not differ from any of the mentioned strains. A coaggregation study was performed for four LAB strains with the pathogen *E. coli* ATCC 10536; the results are presented in Figure 4.

The results (Figure 4) indicate that all LAB strains showed coaggregation with *E. coli* ATCC 10536, reaching a value above 50%. The highest coaggregation was obtained for *L. salivarius* AN9 (61.84% ± 5.70%) and the lowest for *L. plantarum* 47 (53.85% ± 6.30%) and OK-B (53.75 ± 6.60%). Statistical analysis showed differences between LAB coaggregation with *E. coli* ATCC 10536 for *p* < 0.05 (simple classification). Below, Table 3 presents statistically significant differences between LAB strains and *E. coli* ATCC 10536. It was also examined between which pairs of “LAB/pathogen” differences occurred (Table 4).

The statistical analyses indicate differences between specific “LAB/pathogen” pairs. They show that each tested LAB strain showed coaggregation with the tested pathogen.

**Figure 5:**
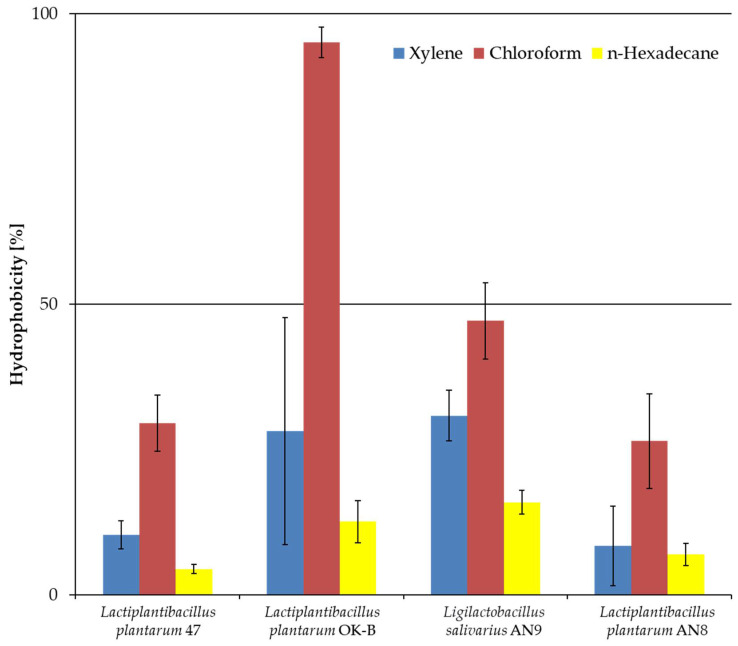
Hydrophobicity of LAB towards three different organic solvents. The experiment was performed in three independent replicates and results are presented as mean ± standard deviation.

Figure 5 shows the degree of hydrophobicity (in %) for four LAB strains in tests with xylene, chloroform, and n-hexadecane. Only *L. salivarius* AN9 achieved the highest affinity for xylene. *L. plantarum* OK-B showed the highest affinity for chloroform at 95.12% ± 2.63%. Each tested strain showed low hydrophobicity (below 20%) to n-hexadecane.

Statistical analysis showed differences between LAB strains, as presented in Table 5 parts a, b, and c. Hydrophobicity results towards xylene for *L. salivarius* AN9 showed statistically significant differences (*p* < 0.05) with *L. plantarum* 47 and AN8. The results of hydrophobicity to chloroform for *L. salivarius* AN9 did not show any statistically significant differences (*p* < 0.01) between all LAB strains. The results of hydrophobicity to n-hexadecane for *L. plantarum* OK-B showed no statistically significant differences between all LAB strains.

### 3.3. Survival of LAB Strains in Unfavorable Gastrointestinal Conditions—In Vitro Test

In these experiments, the survival of LAB strains was determined in the unfavorable conditions of the digestive tract (in vitro) in the presence of bile salts (Table 6) and low pH (Table 7). The concentration of bile salts and the pH value were determined based on the analysis of the literature data on the conditions prevailing in the digestive tract of chickens for fattening.

As a result of the conducted analyses, it was observed that *L. plantarum* 47 showed the highest survival of 54.67% ± 10.19 in bile salt solutions; the lowest viability (26.28% ± 44.52) was recorded for *L. plantarum* AN8 after 4 h of exposure. For two of four strains, the viability was above 50% after 4 h of incubation. Statistical significance was demonstrated between the tested strains and the control (paired *t*-Student’s test, *p* < 0,01).

Table 7 shows the effect of pH (equal to 2.0) on the viability of LAB during 1, 2, and 4 h of exposure to the agent.

The experiment showed a negative effect of low pH on all tested LAB strains after 4 h of incubation. The highest viability (i.e., over 38% after 1 h of incubation) was observed for *L. plantarum* OK-B. No survival at low pH for *L. salivarius* AN9 excluded this strain as a potential probiotic for chickens for fattening. Statistical significance was demonstrated between the tested strains and the control (paired *t*-Student’s test, *p* < 0.01).

### 3.4. The Content of Lactic Acid in Cell-Free Supernatants After LAB Cultivation in MRS Medium and Pasteurized Whey Medium

**Figure 6:**
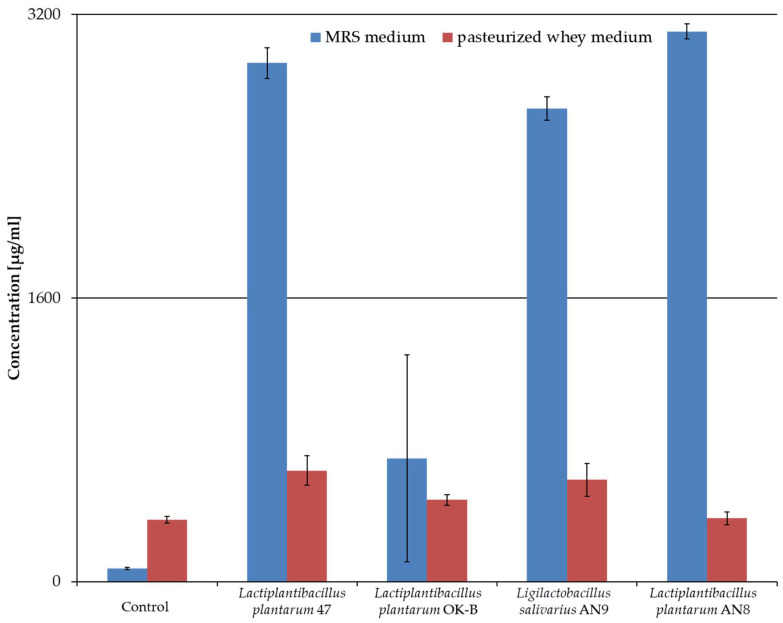
Comparison of lactic acid content determined in cell-free supernatants after culturing LAB in MRS medium and pasteurized whey medium. The controls were pure MRS medium and pasteurized whey medium. The experiment was performed three times, and results are presented as mean ± standard deviation.

The content of lactic acid in CFSs after LAB cultivation in MRS and pasteurized whey medium was determined using the HPLC method. The results are presented in Figure 6.

After fermentation of the MRS medium, lactic acid concentrations ranged from 690 to 3100 µg/mL, depending on the LAB strain. The highest concentrations of this acid compared to the control were observed in CFSs after culturing *L. plantarum* AN8 (3103.83 µg/mL). The statistical analysis performed using the post hoc Dunn’s test showed differences between the acid concentrations in specific samples (*p* < 0.05, Dunn’s test): *L. plantarum* AN8 and control, and *L. plantarum* 47 and control.

Lower lactic acid concentrations were observed in CFS after LAB culture in whey medium than in MRS medium. The control showed a higher concentration of this acid than the MRS control. The results showed that the highest lactic acid concentration was determined in the post-fermentation medium after the *L. plantarum* 47 culture (625.22 µg/mL). Statistical analysis using Dunn’s post hoc test (*p* < 0.05) showed no differences between the concentrations of this acid. The two-sample *t*-test (*p* < 0.05) showed differences between the acid concentration in different post-fermentation MRS media and pasteurized whey media: the control, *L. plantarum* 47 and AN8, and *L. salivarius* AN9.

## 4. Discussion

The experiments determining the probiotic potential of LAB strains in this study aimed to characterize *L. plantarum* 47, AN8, and OK-B, and *L. salivarius* AN9. Probiotic bacteria should exhibit health-promoting effects on the host organism. The application of *Lactiplantibacillus plantarum* CCTCC M2016259 and *Paenibacillus polymyxa* CGMCC1.1711 alleviated the damage to intestinal structures and restored the balance of intestinal microbiota after *Clostridium perfringens* infection. Additionally, body weight gain (BWG) and FCR indices were improved [51,52]. In an experiment conducted by He et al. (2021), the use of *Enterococcus faecium* PNC01 inhibited the multiplication of *Salmonella* Typhimurium, the elongation of intestinal villi was visible, and the multiplication of *Firmicutes* and *Lactobacillus* spp. was increased [53]. First, the bacteria were characterized by their ability to adhere to the intestinal epithelium, high survival in the gastrointestinal tract of humans and animals, support of digestive and metabolic processes, maintenance of the balance of the intestinal microbiome, and stimulation of the host’s immune response [54,55]. The use of sweet whey powder, which is a by-product of cheese production, as the main component of the culture medium allows for a biopreparation to be obtained by partially implementing a closed-loop economy [56]. This substrate contains lactose metabolized by the intestinal microbiota of chickens, improving it and reducing the pH of the intestinal content. Whey proteins also improve the absorption and digestibility of minerals, increase feed intake, and modulate the cecal microbiota, which increases the growth rate of chickens [56,57,58,59,60,61]. One of the ingredients of whey medium is yeast extract. In poultry farming, prebiotic yeast extract is used as a feed additive [23]. Yeast extract has improved BWG and FCR indices and chicken performance. It has also been proven to positively affect the elongation of intestinal villi and reduce crypt depth [62,63].

The first stage of the study was to determine the adhesion of LAB to the abiotic (polystyrene) and biotic (collagen, mucus, and Caco-2 cell line) surfaces. The use of polystyrene in the study is related to the frequent use of this surface as a model for studies on bacterial adhesion [64]. All tested LAB strains showed weak adhesion to polystyrene, a hydrophobic surface. These results may be related to the more hydrophilic nature of LAB strains, which was confirmed by low hydrophobicity tested using n-hexadecane. On the other hand, in the studies by Sepová et al. (2018), the bacteria’s ability to adhere to polystyrene was demonstrated regardless of the surface charge of the cell membrane [65]. Balcazar et al. (2007) reached similar conclusions [66]. In our study, only *L. salivarius* AN9 showed weak adhesion to collagen. All tested *L. plantarum* strains obtained weak adhesion to mucus. The study of the adhesion of potential probiotics to the Caco-2 cell line is one of the main directions for determining the quality of a potential probiotic. The selected LAB strains showed strong adhesion to the Caco-2 cell line. The strongest adhesion was observed for *L. salivarius* AN9 (91.95% ± 1.34%) and the weakest for *L. plantarum* 47 (85.34% ± 1.38%). Barzegar et al. (2021) showed adhesion of *L. plantarum* B20 at 8% [44]. Other studies have shown the adhesion of *L. plantarum* L15 at 12% [67] and *L. plantarum* ŁOCK 0860 at above 92% [68]. These diverse results may indicate that the adhesion abilities of LAB are a species or even strain feature and depend on many exogenous factors.

The study of autoaggregation and hydrophobicity of LAB strains is an important element in determining the effective colonization of probiotics in animal intestines. Strong autoaggregation and hydrophobicity indicate the possibility of adhesion to the intestinal epithelium [69,70,71]. The autoaggregation of probiotic bacteria enables genetic exchange and strengthening of the immune response of the intestinal mucus [72]. On the other hand, coaggregation is an important element of pathogen elimination by generating an unfriendly microenvironment within the pathogen, making it challenging to colonize the gastrointestinal tract [73,74]. The highest autoaggregation value was obtained for the *L. salivarius strain* AN9 (approximately 84%), similar to the study by Xu et al. (2022). *L. salivarius* CML352 showed 84.70% autoaggregation [75]. In other studies, the autoaggregation value of *L. salivarius* was lower, at about 56–57% [76,77]. In this study, the lowest autoaggregation was shown by *L. plantarum* OK-B (about 40%). The remaining strains were characterized by autoaggregation above 50%. The results of analyses obtained by different research teams showed different results for *L. plantarum*, but as a rule, autoaggregation was at a low level: *L. plantarum* in the range from 18.10 to 30.64% [78], VKPM B-11007 8% [76], B20 above 30% [44], and L15 44% [67]. Tuo et al. (2013) conducted studies on the aggregation and adhesion of selected *Lactobacillus* species which were treated with guanidinium chloride, causing a decrease in the ability to autoaggregate and adhere. This indicated the involvement of proteins associated with the bacterial cell surface and other macromolecules in the ability to autoaggregate and adhere [79].

In contrast to autoaggregation, coaggregation forms a bacterial barrier that prevents colonization and biofilm formation by pathogenic bacteria [80]. The results of the coaggregation experiment of LAB strains with *E. coli* ATCC 10536 differed from those described in the literature. *L. plantarum* strains showed a coaggregation value above 50%, while in the literature, it was much lower: *L. plantarum* L15—32% [67], B20—about 30% [44] and *L. plantarum*—less than 5% [81]. A different result was obtained in the work of Nallala et al. (2017), where *L. plantarum* displayed coaggregation with *E. coli* at 62.2 ± 1.03% [82]. Only for the *L. salivarius* strain SML352 was it 47.12%, and for CML350, it was 48.42%, which were at a level similar to the results obtained in the current study [75].

Autoaggregation and hydrophobicity play an important role in the initial contact between a bacterial strain and the host cell [83]. Microbial adhesion to solvents (MATH) tests assess the hydrophobic or hydrophilic properties of bacterial cell surfaces to determine their colonization ability [84,85]. Differences in hydrophobicity testing may be due to the heterogeneous bacterial cell surface, composed of hydrophobic amino acids, lipids, cytoplasmic membrane proteins, surface matrix proteins, and polysaccharides. Hydrophobicity may also be influenced by the cell growth phase and environmental factors [83]. The solvents used in this work represented the following groups: xylene and n-hexadecane, as nonpolar solvents, determined the hydrophobic nature of the bacterial cell membrane; and chloroform, being a polar solvent, described the electron acceptor properties of the cell wall [86]. The value of hydrophobicity towards xylene for *L. salivarius* AN9 was 31% and that for *L. plantarum* 10 was 28% (47, AN8, and OK-B). In the studies, for *L. salivarius,* this affinity was at the level of 61.16% [77] and, for *L. plantarum,* 26.67–91.67% [78]. Another polar solvent was chloroform. The highest hydrophobicity was obtained for *L. plantarum* OK-B (95.12%), which indicates a positive charge of the bacterial membrane. In contrast, the studies conducted by Dell’Anno using *L. plantarum* showed about 50% affinity for chloroform [81]. The last solvent tested was n-hexadecane, for which each LAB strain showed an affinity of less than 20%.

Another issue that has been studied during the typing of potentially probiotic bacteria is survival in the unfavorable conditions of the gastrointestinal tract. Tolerance to bile salts and low pH is a desirable feature of LAB strains. It has been suggested that tolerance to acids and bile salts varies depending on the strain [87]. Isolates from yogurts or dairy products show better adaptation to low pH than isolates obtained from vegetables or meats due to lactose content in the environment, which is converted into lactic acid after fermentation [88,89]. Taking into account the conclusions of Billah et al. (2010) and Ashraf and Smith (2015), studies should be extended to verify the protective effect of whey medium on the viability of LAB strains in bile salts and low pH [88,89]. During exposure to bile salts, cellular homeostasis is disturbed due to the dissociation of the lipid bilayer and the membrane integrity, resulting in the outflow of bacterial cell contents and, thus, their death [90]. In the studies conducted in this paper, the highest tolerance to 0.3% bile salt concentration was demonstrated by the *L. plantarum* strain 47, reaching almost 55% survival after 4 h. The study conducted by Prete et al. (2020) showed excellent tolerance to bile salt stress [91]. Other results for *L. salivarius* in the literature vary. *L. salivarius* can be distinguished by their viability in the presence of bile salts which may be high (even above 62%), medium (in the range of 30.81–66.64%), or very low (below 4%) [75,77,92,93,94]. Differences in the viability of the strains may be due to differences in the expression of proteins involved in cell wall synthesis [95]. *Lactobacillus* tolerates bile salts due to the action of hydrolase reducing the side effects of bile salts. Certain food components can protect and promote strain resistance to bile salts [96]. As in the case of bile salts, the survival of LAB at low pH was varied and dependent on both the strain and the source of the strain. Bujnakova et al. (2014) showed that low pH was toxic to *Lactobacillus* because, after 1 h of exposure to pH 2.0, no viability was observed for 20 tested strains [97]. In the studies conducted in this paper, all four strains did not show viability after 4 h of exposure in the presence of pH 2.0. *L. plantarum* and *L. salivarius* after 3 h at pH 2.0 showed a low survival rate of below 0.001% [93]. However, many studies have reported the survival rate of *L. salivarius* to be above 57% after 5 h, above 80% after 2 h, and generally with good tolerance to low pH [75,77,92,94]. Resistance to low pH stress is described as a specific protein membrane produced by bacteria characterized by tolerance to an acidic environment [95].

LAB produce secondary metabolites with antibacterial activity, including hydrogen peroxide, organic acids (e.g., lactic, fumaric, citric, malic, acetic, and propionic), diacetyl, carbon dioxide, acetoin, acetaldehyde, ammonia, polysaccharides, and ethyl alcohol [98,99,100]. Diacetyl is formed during the metabolism of citrate by LAB such as *Lacticaseibacillus rhamnosus*, *L. plantarum, Lactococcus lactis,* and *Limosilactobacillus fermentum* [101,102,103]. This substance shows antibacterial activity against some Gram-negative bacteria [104]. In our studies, lactic acid was determined after culturing the strains *L. plantarum* AN8, OK-B, and 47, and *L. salivarius* AN9. Studies conducted by Leska et al. (2023) showed the production of lactic acid in the supernatant by all analyzed LAB strains [105]. Hu et al. (2019) reported the production of mainly lactic acid by *L. plantarum*, which showed an inhibitory effect on the growth of *E. coli* and *Salmonella* spp. [106]. Lactic acid has been shown to strengthen the intestinal barrier and facilitate regulating bowel movements in the host [107,108]. Benbara et al. (2020) showed that most bacterial isolates from chicken feces inhibited the growth of *E. coli* ATCC25922 and SL2016 and *Salmonella enterica* CIP 81-3. After neutralization of the supernatant, the antimicrobial activity was lost, which indicates that lactic acid is the main factor inhibiting pathogens [14]. The use of whey medium in this studies decreased lactic acid production compared to the cultivation of the same strains in the MRS medium.

## 5. Conclusions

In summary, the characterized LAB strains show promise for further research on the creation of a biopreparation, which will allow us to decide on the optimal form of its administration. Due to the low viability of LAB strains at low pH and with bile salts, the final product will most likely be a paraprobiotic based on inactivated LAB and their secondary metabolites. Future studies should be conducted on the antagonistic effects of biopreparations on the most common pathogens in chicken breeding, such as *E. coli* or *Clostridium perfringens*. Furthermore, biopreparation should be introduced into in vivo studies on chickens for slaughter.

## Figures and Tables

**Table 1:** Statistically significant differences between LAB adhesion values to the Caco-2 cell line versus the control sample (paired Student’s *t*-test). The control was the density of bacteria before adhesion to the Caco-2 cell line. “+” denotes the occurrence of differences.

	*L. plantarum* 47	*L. plantarum* OK-B	*L. salivarius* AN9	*L. plantarum* AN8
Control	+	+	+	+
First order error	0.01 < *p* < 0.001	0.01 < *p* < 0.001	0.01 < *p* < 0.001	0.01 < *p* < 0.001

**Table 2:** Statistically significant differences between the autoaggregation of LAB strains (simple classification followed by Tukey’s test, *p* < 0.01). “+” denotes the occurrence of differences between strains. “-” denotes no occurrence of differences between strains.

	*L. plantarum* 47	*L. plantarum* OK-B	*L. salivarius* AN9	*L. plantarum* AN8
*L. plantarum* 47	0	-	+	-
*L. plantarum* OK-B	-	0	+	-
*L. salivarius* AN9	+	+	0	-
*L. plantarum* AN8	-	-	-	0

**Table 3:** Statistically significant differences between the coaggregation of LAB strains and *E. coli* ATCC 10536 (paired Student’s *t*-test). “+” denotes the occurrence of differences.

	*L. plantarum* 47	*L. plantarum* OK-B	*L. salivarius* AN9	*L. plantarum* AN8
*E. coli* + LAB ^1^	+	+	+	+
Type I error	0.01 < *p* < 0.001	0.01 < *p* < 0.001	0.01 < *p* < 0.001	0.01 < *p* < 0.001

^1^, lactic acid bacteria.

**Table 4:** Statistically significant differences regarding the occurrence of coaggregation between the pairs “LAB strain and *E. coli* ATCC 10536” (simple classification followed by Tukey’s test, *p* < 0.05). “+” denotes the occurrence of differences between strain mixtures. “-” denotes no occurrence of differences between strain mixtures.

	*L. plantarum* 47 + *E. coli*	*L. plantarum* OK-B + *E. coli*	*L. salivarius* AN9 + *E. coli*	*L. plantarum* AN8 + *E. coli*
*L. plantarum* 47 + *E. coli*	0	-	-	-
*L. plantarum* OK-B + *E. coli*	-	0	+	-
*L. salivarius* AN9 + *E. coli*	-	+	0	-
*L. plantarum* AN8 + *E. coli*	-	-	-	0

**Table 5:** Statistically significant differences between hydrophobicity for individual LAB. “+” denotes the occurrence of a difference. “-” denotes no occurrence of a difference. a—xylene (simple classification, followed by Tukey’s test, *p* < 0.05); b—chloroform (Dunn’s test, *p* < 0.01); c—n-hexadecane (Dunn’s test, *p* < 0.01).

(a)				
	*L. plantarum* 47	*L. plantarum* OK-B	*L. salivarius* AN9	*L. plantarum* AN8
*L. plantarum* 47	0	-	+	-
*L. plantarum* OK-B	-	0	-	-
*L. salivarius* AN9	+	-	0	+
*L. plantarum* AN8	-	-	+	0
(b)				
	*L. plantarum* 47	*L. plantarum* OK-B	*L. salivarius* AN9	*L. plantarum* AN8
*L. plantarum* 47	0	+	-	-
*L. plantarum* OK-B	+	0	-	+
*L. salivarius* AN9	-	-	0	-
*L. plantarum* AN8	-	+	-	0
(c)				
	*L. plantarum* 47	*L. plantarum* OK-B	*L. salivarius* AN9	*L. plantarum* AN8
*L. plantarum* 47	0	-	+	-
*L. plantarum* OK-B	-	0	-	-
*L. salivarius* AN9	+	-	0	-
*L. plantarum* AN8	-	-	-	0

**Table 6:** Survival of LAB strains in 0.3% bile salt solutions after 1, 2, and 4 h of incubation at 40 °C. The experiment was performed in three independent experiments and presented as mean ± standard deviation.

Strain	Viability [%] ± SD		
	1 h	2 h	4 h
*L. plantarum* 47	68.47 ± 7.31	56.25 ± 5.53	54.67 ± 10.19
*L. plantarum* OK-B	78.85 ± 5.07	70.32 ± 13.20	35.64 ± 21.23
*L. salivarius* AN9	49.02 ± 5.68	46.78 ± 9.31	50.21 ± 10.06
*L. plantarum* AN8	43.47 ± 23.84	34.05 ± 33.07	26.28 ± 44.52

**Table 7:** Survival of LAB strains in PBS solutions at pH 2.0 after 1, 2, and 4 h of incubation at 40 °C. The experiment was performed in three independent experiments and presented as mean ± standard deviation.

Strain	Viability [%] ± SD		
	1 h	2 h	4 h
*L. plantarum* 47	8.35 ± 173.21	0	0
*L. plantarum* OK-B	38.23 ± 24.77	0	0
*L. salivarius* AN9	0	0	0
*L. plantarum* AN8	20.99 ± 124.14	0.08 ± 173.21	0

## Data Availability

The data presented in this study are available in this article and are available from Daria Zamojska upon reasonable request.
